# Transcranial direct current stimulation combined with caffeine promotes executive function in healthy females

**DOI:** 10.1080/15502783.2025.2555249

**Published:** 2025-09-01

**Authors:** Weiqin Yuan, Michael A. Nitsche, Tian Yue, Ying Yu, Fengxue Qi

**Affiliations:** a Beijing Sport University, Key Laboratory of Sport Training of General Administration of Sport of China, Beijing, China; b Beijing Sport University, Sports, Exercise and Brain Sciences Laboratory, Sports Coaching College, Beijing, China; c Leibniz Research Centre for Working Environment and Human Factors, Department of Psychology and Neurosciences, Dortmund, Germany; d University Clinic of Psychiatry and Psychotherapy, Protestant Hospital of Bethel Foundation, University Hospital OWL, Bielefeld University, Bielefeld, Germany; e German Centre for Mental Health (DZPG), Bochum, Germany; f Shandong University, School of Physical Education, Shandong, China

**Keywords:** Transcranial direct current stimulation, executive function, caffeine, female

## Abstract

**Background:**

Studies have demonstrated that both transcranial direct current stimulation (tDCS) and caffeine improve cognitive abilities through similar mechanisms. This study investigated the acute effects of tDCS combined with caffeine on executive functions.

**Methods:**

Eighty females were randomly assigned to four groups (tDCS + caffeine, tDCS + placebo, sham tDCS + caffeine, and sham tDCS + placebo). Each participant completed two experimental sessions. In the first session, participants performed the Stroop, 3-back, and More-Odd Shifting tasks (T0). For the second session, participants ingested a 200 mg caffeine capsule/placebo, waited 45 minutes, and then received 2 mA real or sham tDCS for 20 minutes. Next, they performed the same cognitive tasks as in the first session (T1), and rested for 30 minutes before completing the cognitive test again (T2).

**Results:**

Accuracy in the 3-back task was significantly improved for the tDCS + caffeine group at T1 and T2, for the tDCS + placebo group at T1, and for the sham tDCS + caffeine group at T2 compared to the sham tDCS + placebo group. Accuracy on the Stroop task was significantly enhanced in the tDCS + caffeine group at T1 compared to the sham tDCS + caffeine and sham tDCS + placebo groups, and in the tDCS + placebo group at T1 compared to the sham tDCS + caffeine group. No significant differences were observed among groups for the More-Odd Shifting task.

**Conclusions:**

These findings suggest that both tDCS and caffeine interventions can improve cognitive task performance, and their combination results in more persistent improvements in executive functions compared to tDCS or caffeine alone.

## 1. Introduction

Executive functions (EFs) are crucial for managing many aspects of life, and individuals with stronger EFs are more likely to succeed academically and professionally. EFs refer to the higher cognitive abilities required to regulate various mental processes to achieve specific goals [1]. The primary components of EFs include working memory, inhibitory control, and cognitive flexibility [2]. Working memory involves the temporary storage and processing of information during cognitive tasks [3]. Inhibitory control refers to the ability to consciously suppress dominant or automatic responses and avoid interference from irrelevant information [4]. Cognitive flexibility represents the capacity to rapidly and adaptively adjust to shifting circumstances [5]. The neural basis of executive functions is a common cognitive control network that includes the dorsolateral prefrontal cortex (DLPFC), orbitofrontal cortex, anterior cingulate, and frontopolar cortex [6]. Given the importance of EFs and their impairment in neurologic and psychiatric disorders, it is essential to explore techniques and interventions aimed at enhancing them [7].

Transcranial direct current stimulation (tDCS) is a noninvasive and safe neurostimulation method that modulates targeted brain regions by delivering weak direct currents, leading to alterations in cortical excitability [8]. Anodal stimulation increases excitability by depolarizing cortical neurons, while cathodal stimulation decreases it by hyperpolarizing neuronal membranes. The changes in cortical excitability induced by tDCS occur during stimulation and continue after stimulation has ceased, provided that the intervention is both sufficiently long and strong [9]. By altering the cortical excitability of the stimulated brain area and strengthening connections between different brain areas, tDCS can improve cognitive functions, such as working memory [10] and inhibitory control [11], in healthy individuals. Studies have combined tDCS with other interventions to investigate potentially complementary or additive relationships [12,13], though knowledge in this field is incomplete.

Caffeine is the most widely used central nervous system stimulant worldwide. It is predominantly ingested through coffee and tea, although it is present in various other beverages and foods [14]. Caffeine has a similar structure to adenosine, a neuromodulator that plays a role in inhibiting the release of various neurotransmitters. Caffeine acts as an antagonist to adenosine A1 and A2A receptors, effectively blocking them, thus mitigating the inhibitory effects of adenosine and facilitating an increase in neurotransmitter release [15]. Furthermore, caffeine regulates GABAergic synapses by inhibiting GABAergic neurotransmission [16,17]. Caffeine is also thought to promote neurotransmitter release and enhance cortical excitability through the interplay of multiple mechanisms, thereby improving various cognitive functions including memory, attention, and cognitive flexibility [18,19].

Anodal tDCS and caffeine, individually as well as in combination, have been shown to enhance muscle power and training volume in men compared to placebo. However, only combined interventions resulted in a lower rating of perceived exertion (RPE) compared to placebo, with alterations in cortical excitability posited as a key factor influencing this reduction [20,21]. Both tDCS and caffeine enhance cognitive function by altering cortical excitability, because of their similar physiological effects, synergies are expected in their combined intervention. In addition, the effects of single-session tDCS and caffeine interventions on cognitive function are influenced by various factors such as stimulation parameters, dosage, and sex. Sex differences have been observed in the impact of caffeine, with a greater effect observed in males compared with females [22]. Previous studies on combined interventions only included male participants, highlighting the need for further investigation.

This study aimed to explore the impact of tDCS over the left DLPFC combined with caffeine on executive functions in females. We hypothesized that individual tDCS and caffeine interventions would lead to significant improvements in EFs and that tDCS combined with caffeine would improve EFs beyond the level achieved by each intervention alone.

## 2. Methods

### 2.1. Participants

Eighty healthy participants from Beijing Sport University took part in the experiments after providing written informed consent. Participants were randomly assigned equally to four groups (tDCS + caffeine, tDCS alone, caffeine alone, sham tDCS + placebo) via a simple random assignment. All experimental procedures were reviewed and approved by the Ethics Committee for Human Experiments at Beijing Sport University (2023157 H) and adhered to the principles outlined in the Declaration of Helsinki. The inclusion criteria for participation were as follows: (a) age between 18 and 35 years and right-handedness; (b) absence of color blindness, color weakness, or dyslexia; (c) normal or corrected-to-normal vision; (d) no pregnancy, cardiovascular disease, or other physical impairments, and no personal or family history of mental illness or substance abuse; (e) absence of any internal or external medical device implants; (f) caffeine intake of less than 150 mg per day (or abstention) and being a nonsmoker [23]. See Table 1.

**Table 1.** Demographic characteristics of all participants.

Variable	M ± SD
Age (years)	19.34 ± 1.01
Height (cm)	162.99 ± 4.88
Weight (kg)	55.63 ± 8.93
BMI (kg/$m^{2}$)	20.93 ± 3.16

Note: BMI, body mass index.

### 2.2. Experimental tasks

#### 2.2.1. 3-back working memory task

**Figure 1.**
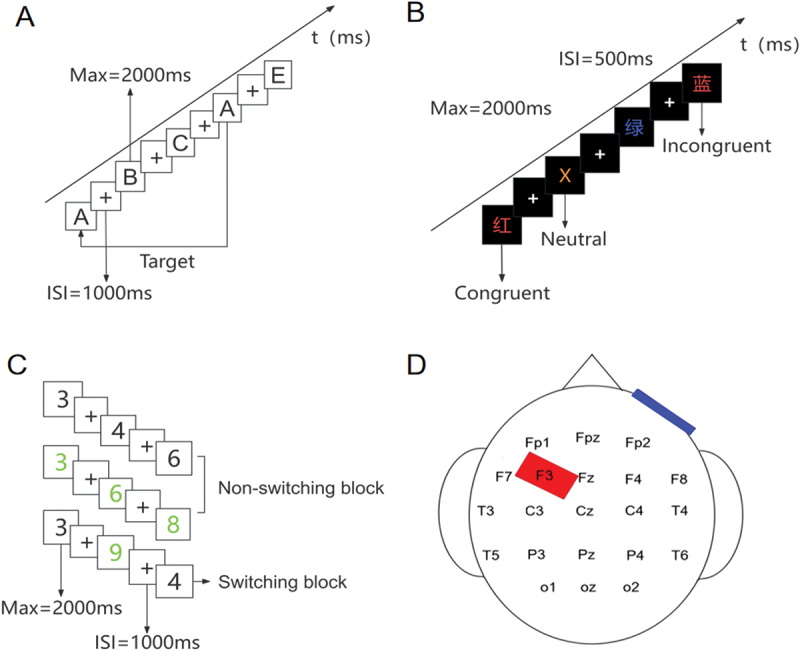
(A) The 3-back task. Participants were required to identify whether the presented letter matched the one shown three trials earlier. (B) The Stroop task. Participants were instructed to indicate the color of the ink. (C) The more-Odd shifting task. Participants were required to adjust their responses according to different rules indicated by the color of the presented number. (D) tDCS intervention. The anodal electrode was positioned over the left DLPFC, while the cathodal electrode was placed over the contralateral supraorbital region.

The 3-back task was used to assess working memory. Participants performed the task while seated in a chair, placed at a comfortable distance from a 17-inch computer screen. They were given a verbal explanation of the rules of the task and allowed to practice in order to familiarize themselves with the task. A series of letters from A to J were presented on the screen in a pseudorandom order. Each letter was displayed for 2000 ms in between the 500-ms presentation of a blank screen. There was a total of 155 trials, including 27 practice trials, 32 matched trials, and 96 mismatched trials. Participants were required to identify whether each letter corresponded to the one displayed three positions earlier in the sequence. Participants responded matched or “mismatched” by pressing the “V” or “N” keys on a keyboard as quickly and accurately as possible. Completing the task required roughly 8 min. The task was implemented using the E-Prime Software Version 3.0. Accuracy (ACC) and reaction time (RT) were utilized as behavioral metrics for this task. See Figure 1(A).

#### 2.2.2. Stroop task

The Stroop task was performed to measure inhibitory control. A string of Chinese characters (红、蓝、黄、绿) and the letter X were displayed in one of four colors (yellow, blue, yellow, green) on a computer screen. Participants were instructed to identify the color by quickly pressing the corresponding keys on the keyboard, with D, F, J, and K representing red, green, yellow, and blue, respectively. In the congruent condition, the meaning and color of the Chinese characters were consistent. In the incongruent condition, the meaning and color of the Chinese characters were inconsistent. In the neutral condition, “X” was randomly presented in different colors. The task consisted of 144 trials, with 48 trials under each of the different conditions (congruent, neutral, and incongruent). Each stimulus was displayed for 2000 ms, followed by a 500 ms inter-stimulus interval. Participants received a verbal explanation of the task rules and were provided with practice time, with the practice block comprising 18 trials. The screen was positioned at an optimal viewing distance to ensure participant comfort. The entire task took approximately 7 minutes to complete, with ACC and RT recorded as behavioral outcome measures. See Figure 1(B).

#### 2.2.3. More-Odd shifting task

The More-Odd Shifting Task was used to evaluate cognitive flexibility [24,25]. Participants were required to adapt their responses based on varying rules. A series of numbers (1–4, 6–9) were presented in the center of the screen in either black or green. When a black number was displayed on the screen, participants were tasked with determining whether it was greater or less than 5, with the F key corresponding to numbers less than 5 and the “J” key to those greater than 5. When a green number appeared, participants were required to evaluate its parity, pressing “F” for odd and “J” for even numbers. The task comprised three blocks: block A with all numbers in black, block B with all numbers in green, and block C with a mix of black and green numbers presented randomly. Blocks A and B comprised 16 trials each, and block C included 32 trials. Additionally, blocks A and C each included eight practice trials, whereas block B contained 16 practice trials. The sequence of blocks followed the order “ABCCBA.” Each number was shown on a 17-inch computer screen with a white background for 2000 ms with a 1000 ms interval between numbers. The screen was set at an optimal distance to facilitate comfortable viewing. The entire task took approximately 8 minutes to complete. RT, ACC, and switch cost served as behavioral measures, with switch cost calculated as the difference in reaction time between the switching block (block C) and the non-switching blocks (blocks A and B). See Figure 1(C).

#### 2.2.4. Transcranial direct current stimulation

Direct current was administered via saline-soaked surface sponge electrodes (35 cm^2^) using a battery-operated constant current stimulator (TCS-E2000, Shenzhen Yingchi Technology Co., Ltd). The ramping up and ramping down duration of the direct current at both the onset and conclusion of stimulation was set to 15 seconds. Participants were randomly assigned to receive either anodal stimulation over the left DLPFC or sham tDCS. The anodal electrode was positioned over the left DLPFC at the F3 location based on the International 10–20 electrode system, while the cathodal electrode was positioned over the contralateral supraorbital region. For real stimulation, the current intensity was set to 2 mA for a duration of 20 minutes. In the sham condition, the current was ramped up and down for 15 seconds at both the start and end to simulate the sensation of active stimulation. Participants did not know which type of tDCS they were receiving, and they were instructed to sit in a quiet environment during the stimulation period. See Figure 1(D).

### 2.3. Experimental protocol

**Figure 2.**
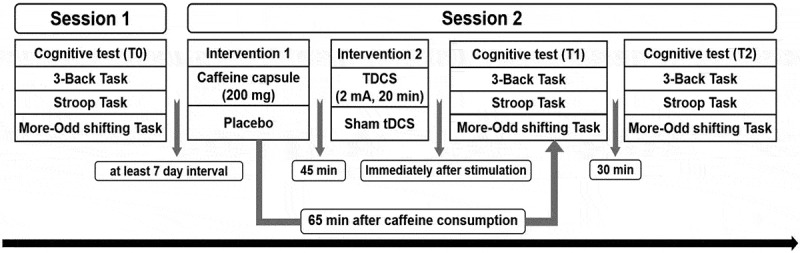
Experimental design.

This study employed a single-blind, parallel-group, sham-controlled design. Participants were randomized to four different interventions, and each participant completed two experimental sessions with an interval of at least 1 week to eliminate the practice effect from the first session [26]. Upon their initial visit to the laboratory, participants received a detailed briefing on the experimental procedures and were administered questionnaires for assessing demographics and obtaining written informed consent. Subsequently, the Stroop, 3-back, and More-Odd shifting tasks were conducted in randomized order lasting approximately 24 minutes (T0), with breaks allowed between each cognitive task. In the second session, participants consumed a capsule that either contained caffeine or served as a placebo and then waited for 45 minutes to allow the drug to be absorbed. Salivary caffeine levels were observed to peak at approximately 67 ± 7 minutes post-ingestion [27]. Participants were permitted to read to maintain wakefulness during the waiting period. After waiting, participants received tDCS over the left DLPFC or sham tDCS for 20 min, followed by the three cognitive tasks described above in randomized order (T1). Participants were allowed to rest for 30 minutes after completing the tasks before performing them again (T2). See Figure 2.

### 2.4. Statistical analysis

For the 3-back task, incorrect responses and reaction times under 200 ms were excluded as outliers [28]. For the Stroop Color-Word task, incorrect responses and RTs of less than 100 ms that were more than three standard deviations (3 SDs) from the mean were excluded as outliers. ACC and RTs were analyzed separately for the different conditions (congruent, neutral, and incongruent) [29,30]. For the More-Odd shifting task, incorrect responses and RTs exceeding 3 SDs from the mean were excluded as outliers. ACC and RTs were assessed independently for distinct conditions (non-switching, switching). Two-way repeated measures analyses of variance (ANOVA) were conducted, with time (three time points: T0, T1, T2) as the within-subjects factor and group (tDCS + caffeine group, tDCS alone group, caffeine alone group, sham tDCS + placebo group) as the between-subjects factor. Mauchly’s test was applied to check for sphericity, and when the assumption was violated, the Greenhouse – Geisser correction was used. Post-hoc least significant difference (LSD) comparisons were conducted when the ANOVA results indicated significant differences. Statistical significance was determined at *p* < 0.05. Partial eta squared ($\eta_{p}^{2}$) served as the indicator of effect size. All statistical analyses were executed using SPSS version 26.

## 3. Results

### 3.1. 3-back task

**Figure 3.**
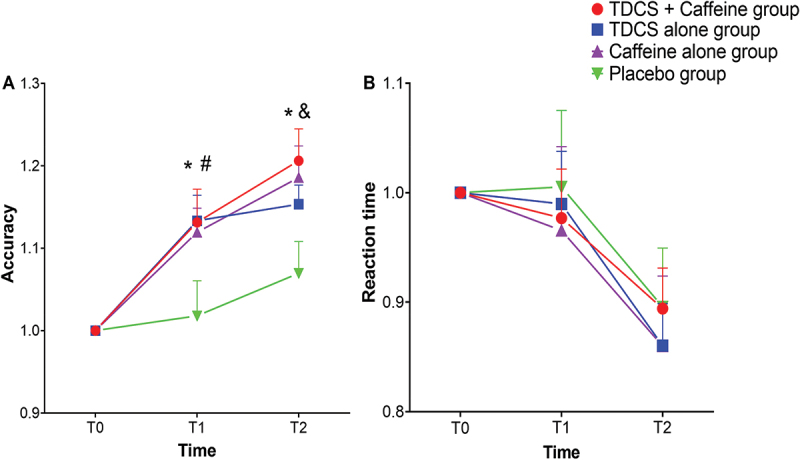
Results of various interventions on accuracy rate (A) and reaction time (B) of the 3-back task. * denotes the significant differences (*p* < 0.05) between the tDCS + caffeine group and sham tDCS + placebo group; # denotes the significant differences (*p* < 0.05) between the tDCS and sham tDCS + placebo groups; & denotes the significant differences (*p* < 0.05) between the caffeine alone and sham tDCS + placebo groups. Error bars represent the SEM. tDCS, transcranial direct current stimulation. Placebo group, sham tDCS + placebo group.

For ACC, the repeated measures ANOVA revealed a significant main effect of time (*F*_(1.364, 103.698)_ = 50.956, *p* < 0.001, $\eta_{p}^{2}$ = 0.401) and a significant interaction between group and time (*F*_(4.093, 103.698)_ = 2.530, *p* = 0.044, $\eta_{p}^{2}$ = 0.091) but no significant main effect of group (*F*_(3, 76)_ = 2.650, *p* = 0.055, $\eta_{p}^{2}$ = 0.095). ACC significantly improved at T1 (*p* = 0.030) and T2 (*p* = 0.008) in the tDCS + caffeine group compared to the sham tDCS + placebo group. A significant increase in ACC was observed at T1 in the tDCS alone group compared with the sham tDCS + placebo group (*p* = 0.027), and a significantly higher ACC was found in the caffeine alone group at T2 relative to the sham tDCS + placebo group (*p* = 0.023). However, no significant differences were demonstrated between the combined intervention and the caffeine-alone group or the tDCS-alone group at all times point (*p* > 0.05). See Figure 3A.

For RT, a significant main effect of time was found (*F*_(1.353, 102.824)_ = 14.942, *p* < 0.001, $\eta_{p}^{2}$ = 0.164), but the effect of group (*F*_(3, 76)_ = 0.089, *p* = 0.966, $\eta_{p}^{2}$ = 0.004) and the interaction between group and time (*F*_(4.059, 102.824)_ = 0.149, *p* = 0.964, $\eta_{p}^{2}\;$ = 0.006) were not significant. See Figure 3B.

### 3.2. Stroop color-word task

**Figure 4.**
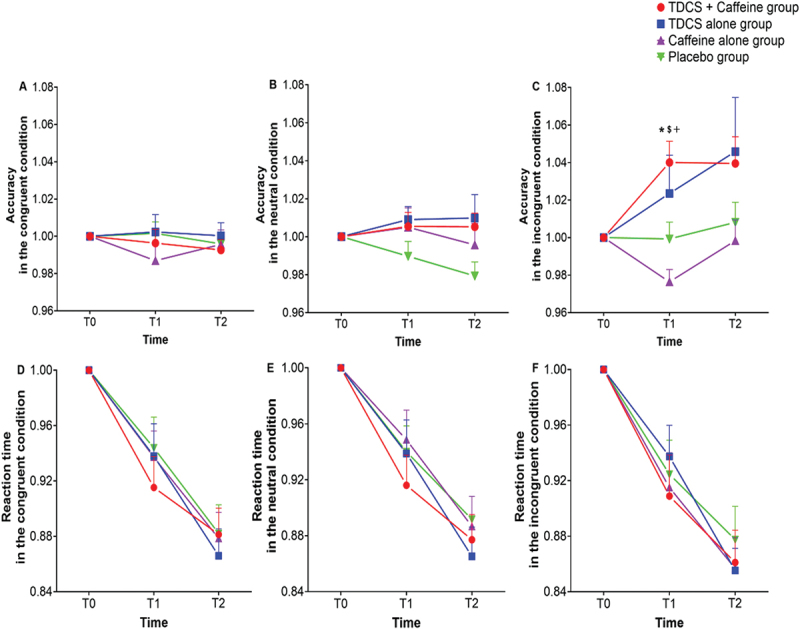
Results of different interventions on accuracy rate (A, B, C) and reaction time (D, E, F) of the Stroop color-word task are shown. A and D reflect task performance in the congruent condition; B and E reflect task performance in the neutral condition; C and F reflect task performance in the incongruent condition. * indicates significant differences (*p* < 0.05) between the tDCS + caffeine and sham tDCS + placebo group; $ indicates significant differences (*p* < 0.05) between the tDCS + caffeine and caffeine alone groups; + indicates significant differences (*p* < 0.05) between the tDCS alone and caffeine alone groups. Error bars represent the SEM. tDCS, transcranial direct current stimulation. Placebo group, sham tDCS + placebo group.

For ACC, the repeated measures ANOVA indicated significant main effects of time (*F*_(1.400, 106.423)_ = 5.443, *p* = 0.012, $\eta_{p}^{2}$ = 0.067), group (*F*_(3, 76)_ = 2.901, *p* = 0.040, $\eta_{p}^{2}$ = 0.103), and interaction between group and time (*F*_(4.201, 106.423)_ = 2.459, *p* = 0.047, $\eta_{p}^{2}$ = 0.088) in the incongruent condition. ACC in the tDCS + caffeine group was higher at T1 relative to the caffeine alone group (*p* = 0.01) and the sham tDCS + placebo group (*p* = 0.028). Furthermore, the tDCS alone group showed higher ACC at T1 in comparison with the sham tDCS + placebo group (*p* = 0.012). The main effects of time (*F*_(2, 152)_ = 0.643, *p* = 0.527, $\eta_{p}^{2}$ = 0.008), group (*F*_(3, 76)_ = 0.492, *p* = 0.689, $\eta_{p}^{2}$ = 0.019), and interaction (*F*_(6, 152)_ = 0.633, *p* = 0.704, $\eta_{p}^{2}$ = 0.024) were not significant in the congruent condition. Similarly, the main effects of time (*F*_(2, 152)_ = 0.598, *p* = 0.551, $\eta_{p}^{2}$ = 0.008), group (*F*_(3, 76)_ = 1.830, *p* = 0.149, $\eta_{p}^{2}\;$ = 0.067), and the respective interaction (*F*_(6, 152)_ = 1.273, *p* = 0.273, $\eta_{p}^{2}$ = 0.048) were not significant in the neutral condition. See Figures 4A,B,C.

For RT, we observed a significant main effect of time (*F*_(1.559, 118.450)_ = 84.025, *p <* 0.001, $\eta_{p}^{2}$ = 0.525) in the incongruent condition, but no significant changes in the main effect of group (*F*_(3, 76)_ = 0.125, *p* = 0.945, $\eta_{p}^{2}$ = 0.005) or in the interaction between group and time (*F*_(4.676, 118.450)_ = 0.387, *p* = 0.845, $\eta_{p}^{2}$ = 0.015). In the congruent condition, a significant main effect of time (*F*_(1.759, 133.661)_ = 83.062, *p <* 0.001, $\eta_{p}^{2}$ = 0.522), but no significant main effect of group (*F*_(3, 76)_ = 0.114, *p* = 0.952, $\eta_{p}^{2}$ = 0.004) or the interaction between group and time (*F*_(5.276, 133.661)_ = 0.433, *p* = 0.834, $\eta_{p}^{2}$ = 0.017) were found. The two-way repeated measures ANOVA revealed a significant main effect of time (*F*_(1.542, 117.167)_ = 87.196, *p <* 0.001, $\eta_{p}^{2}$ = 0.534), but not of group (*F*_(3, 76)_ = 0.284, *p* = 0.837, $\eta_{p}^{2}$ = 0.011) nor of the respective interaction (*F*_(4.625, 117.167)_ = 0.566, *p* = 0.712, $\eta_{p}^{2}$ = 0.022) in the neutral condition. See Figures 4D, 4E, and Figure 4F.

### 3.3. More-Odd shifting task

**Figure 5.**
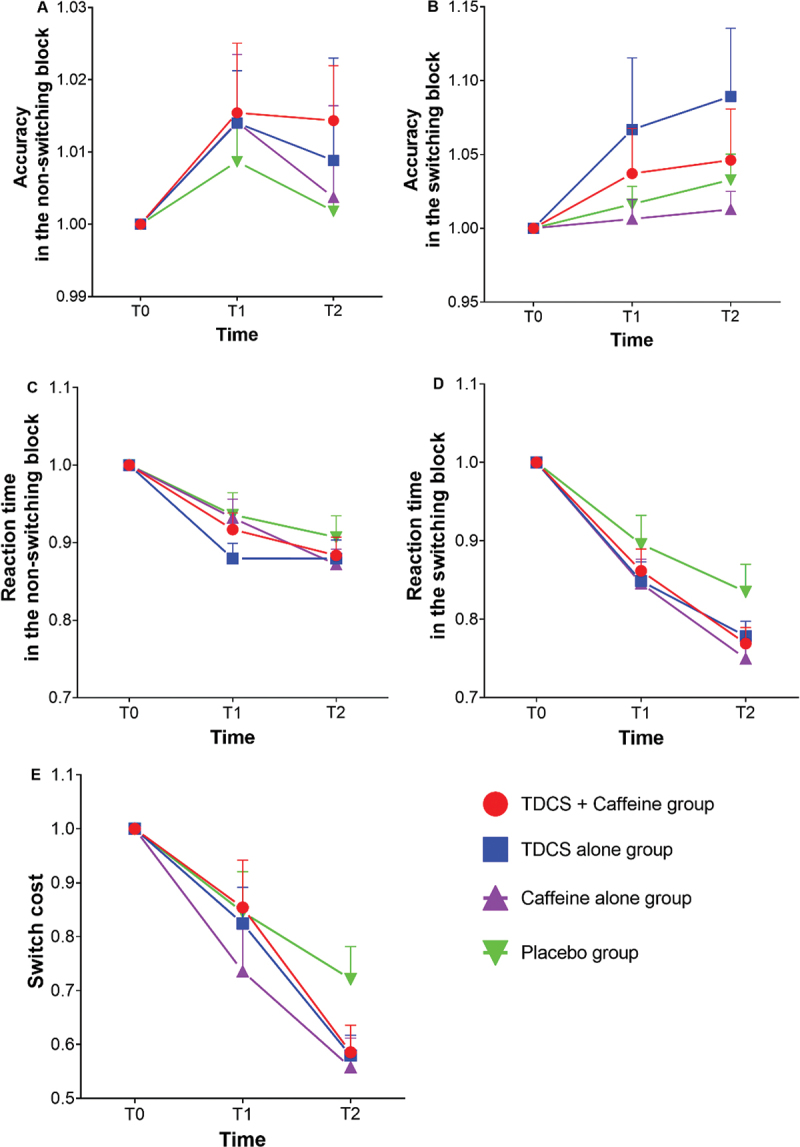
Results of different interventions on mean accuracy rate (A, B) and reaction time (C, D) and switch cost (E) of the more-Odd shifting task. A and C reflect task performance under non-switching conditions; B and D reflect task performance under switching conditions. Error bars represent the SEM. tDCS: transcranial direct current stimulation. Placebo group, sham tDCS + placebo group.

For ACC, there was a significant main effect of time (*F*_(1.838, 139.661)_ = 3.668, *p* = 0.031,$\eta_{p}^{2}$ = 0.046) in the non-switching condition, while the main effects of group (*F*_(3, 76)_ = 0.234, *p* = 0.872, $\eta_{p}^{2}$ = 0.009) or interaction between group and time (*F*_(5.513, 139.661)_ = 0.198, *p* = 0.971,$\eta_{p}^{2}$ = 0.008) were not significant. A significant main effect of time (*F*_(1.383, 105.102)_ = 6.221, *p* = 0.008, $\eta_{p}^{2}$ = 0.076) but not of group (*F*_(3, 76)_ = 1.005, *p* = 0.396, $\eta_{p}^{2}$ = 0.038) nor of the respective interaction (F _(4.419, 105.102)_ = 0.875, *p* = 0.485, $\eta_{p}^{2}$ = 0.033) emerged in the switching condition. See figure Figures 5A, and 5B.

For RT, the results showed significant main effects of time (*F*_(1.819, 138.250)_ = 57.534, *p <* 0.001, $\eta_{p}^{2}$ = 0.431), but not group (*F*_(3, 76)_ = 0.609, *p* = 0.611, $\eta_{p}^{2}$ = 0.023) nor the respective interaction (*F*_(5.457, 138.250)_ = 1.009, *p* = 0.418, $\eta_{p}^{2}$ = 0.038) in the non-switching condition. A significant main effect of time (*F*_(1.754, 133.308)_ = 140.833, *p <* 0.001, $\eta_{p}^{2}$ = 0.650), but not for the main effect of group (*F*_(3, 76)_ = 1.290, *p* = 0.284, $\eta_{p}^{2}$ = 0.048) nor for the respective interaction (*F*_(5.262, 133.308)_ = 1.053, *p* = 0.391, $\eta_{p}^{2}$ = 0.040) were observed in the switching condition. See Figures 5C, and 5D.

For switch cost, the result yielded a significant main effect of time (*F*_(1.675, 127.273)_ = 68.638, *p <* 0.001, $\eta_{p}^{2}$ = 0.475), but no significant effects were observed for group (*F*_(3, 76)_ = 1.007, *p* = 0.394, $\eta_{p}^{2}$ = 0.038) nor the interaction between group and time (F _(5.024, 127.273)_ = 0.971, *p* = 0.438, $\eta_{p}^{2}$ = 0.037). See Figure 5E.

## 4. Discussion

This study examined the effects of tDCS combined with caffeine on EFs. With respect to working memory, the combination of tDCS and caffeine enhanced the accuracy of performance on the 3-back task at T1 and T2 compared with the sham tDCS + placebo group. Additionally, tDCS alone boosted accuracy at T1, and caffeine alone increased accuracy at T2 compared to the sham tDCS + placebo group. In terms of inhibitory control, the combination of tDCS and caffeine significantly improved the accuracy of performance on the Stroop task at T1 compared to the caffeine alone and sham tDCS + placebo groups. Furthermore, tDCS alone significantly increased accuracy on the Stroop task at T1 relative to caffeine alone, but caffeine alone did not produce any enhancements in inhibitory control relative to the other groups. Neither tDCS combined with caffeine nor tDCS or caffeine alone demonstrated significant effects with respect to cognitive flexibility measured using the More-Odd task.

Regarding working memory, the results showed that both single and combined interventions increased the accuracy of 3-back task performance, which is consistent with our hypothesis. Although there were no significant differences in the extent of improvement between single interventions and combined interventions, only the combined intervention improved accuracy at both T1 and T2, indicating that the combined intervention led to improvements persisting for at least 30 minutes in working memory performance. Consistent with the current research findings indicating that tDCS significantly improved the accuracy of the 3 back task at T1, many other studies have demonstrated a positive impact of tDCS on working memory [31,32].

Anodal tDCS, as applied in the present study, can depolarize glutamatergic synaptic membranes, increasing calcium influx and ultimately leading to the strengthening of synaptic connections [33]. However, there was no significant improvement compared to the sham tDCS + placebo group in T2. This is similar to the results of Ohn et al., who reported that anodal tDCS over the left DLPFC enhanced working memory in a time-dependent manner, with effects lasting up to 30 minutes post-stimulation [34]. The stimulation duration in their study was, however, longer (30 minutes) than that in our current study. Besides the stimulation parameters, the cognitive tests at T2 were conducted more than 30 minutes after tDCS, suggesting that this long interval might have contributed to the observed null result for the anodal tDCS alone at T2.

The cognitive effects of caffeine are primarily caused by its ability to block adenosine A1 receptors in the hippocampus and cortex, which are the brain regions most associated with cognitive functions [35]. Adenosine is thought to suppress the release of various neurotransmitters, including norepinephrine, dopamine, serotonin, acetylcholine, and glutamate [36]. Caffeine enhances the release of these neurotransmitters by preventing adenosine from binding to A1 and A2A receptors, thus regulating neuronal excitability [37,38]. In addition, caffeine improved working memory at T2 but had no effect at T1 (65 minutes after caffeine consumption). Peak saliva caffeine levels have been reported to occur at 67 ± 7 minutes after consumption of caffeine capsules; however, the time to reach peak subjective effects in participants is between 85 and 130 minutes [27]. Another study indicated that caffeine improved accuracy in an attention-switching task 90 minutes but not 60 minutes after ingestion [39]. Given the high cognitive demands of the 3-back task and attention-switching tasks, caffeine may require a longer absorption time to affect higher-order cognitive functions. The combination of tDCS and caffeine has been shown to significantly reduce RPE, which is attributed to changes in cortical excitability [20]. Caffeine has been shown to reduce the cortical silent period duration; this effect is likely mediated by GABA receptors, and caffeine may decrease GABA release, thereby reducing GABAergic inhibitory transmission [40,41]. A study utilizing magnetic resonance spectroscopy observed a reduction in GABA levels after tDCS [42]. Therefore, in addition to mediating glutaminergic synapses to enhance cortical excitability, a reduction in GABAergic inhibition may be another potential mechanism underlying the improvement of working memory. In addition, previous studies have shown that caffeine prolonged and enhanced long-term potentiation-like aftereffects induced by paired associative stimulation (PAS 25), despite the differences in intervention methods between tDCS and PAS 25, both can induce synapse-specific plasticity aftereffects by modulating NMDA receptors and voltage-dependent calcium channels [43]. Therefore, caffeine may have contributed to prolonged improvements in cortical excitability induced by tDCS. While reaction time improvements within the group across all groups may reflect practice effects, targeted measures were implemented in the experimental design to address this concern. Randomization of group assignments and task sequences was conducted to reduce potential biases introduced by practice effects. Consequently, practice effects are unlikely to be the primary contributor to the observed enhancements.

The present study revealed no beneficial effects of tDCS or caffeine on inhibitory control. Some studies indicate that both tDCS and caffeine alone can improve inhibitory control, but the effect is influenced by factors such as dosage, paradigm, and sex differences [44–46]. Both unilateral and bilateral tDCS stimulation can enhance inhibitory control abilities. However, some studies suggest that dual-point stimulation is superior to traditional single-point stimulation [47–49]. In this study, the anode electrode was positioned on the left DLPFC, while the cathode electrode was located over the opposite supraorbital region. Consequently, the different stimulation montages may influence the intervention effect of tDCS on inhibitory control. What is interesting is that it was associated with a significant improvement compared to caffeine; this is not entirely unexpected. A study compared the anti-fatigue effects of tDCS and caffeine, and the results showed that tDCS improved task performance on the Psychomotor Vigilance Test (PVT) when applied early in the sleep deprivation period. Additionally, participants who received tDCS felt more energetic and experienced less fatigue [50]. Therefore, tDCS may be more effective in improving cognitive function than caffeine.

Dixit et al. [51] found that caffeine can accelerate the processing of relevant information, as reflected in the changes in RTs to neutral, incongruent, and congruent stimuli in the Stroop task. Another study showed that both 3 mg/kg and 6 mg/kg of caffeine significantly reduced male participants’ RTs to incongruent stimuli in the Stroop task. However, only the 3 mg/kg dose resulted in a reduction of RT to congruent stimuli [52]. While these findings indicate that caffeine can improve inhibitory control, other research has reported that doses of 125 mg and 250 mg did not significantly influence performance on the Stroop task [53]. The similarity between the latter study and our experiment is that both used a fixed dose (200 mg in our experiment) rather than a dose determined based on body weight. Therefore, the relative differences in dosage may lead to the variation in results.

In addition, sex may also influence the effects of caffeine on inhibition control. The above-mentioned studies demonstrating the effects of caffeine in enhancing inhibitory control recruited only male participants, whereas we investigated the cognitive effects of caffeine on a female population. Caffeine has a more pronounced impact on cognitive performance in males compared to females, suggesting that females have a lower sensitivity to caffeine [54]. One study employing caffeine doses of up to 6 mg/kg seems to support this perspective. The findings revealed that caffeine significantly enhanced enjoyment and excitement ratings among female team game participants during intermittent exercise and also increased vigor. However, it only demonstrated a trend toward improved RTs in the Stroop task [55]. Therefore, sex differences may be an additional factor contributing to the variation in experimental results. The combined intervention improved performance in the Stroop task at T1. We suggest that this finding is consistent with the mechanism by which the combined intervention enhances working memory in the current study, namely that caffeine has the potential to both enhance and extend the effects of tDCS on cortical excitability.

With respect to cognitive flexibility, the present results appear to conflict with studies reporting significant effects of tDCS or caffeine on task switching [56–59]. No significant improvements in More-Odd shifting task (switching condition, non-switching condition, and switching cost) were observed following either combined tDCS and caffeine interventions or single interventions. Several factors may contribute to this discrepancy. For tDCS, previous studies predominantly employed cross- hemispheric and online tDCS paradigms to investigate their effects on cognitive flexibility, whereas we employed unilateral and offline tDCS stimulation. Results revealed that healthy participants receiving cross-hemispheric stimulation exhibited significantly enhanced task-switching ability, accompanied by asymmetric activation patterns in the frontoparietal network, which suggests that merely increasing cortical excitability may not suffice to improve cognitive flexibility [60]. Moreover, task switching under switching conditions mainly relies on exogenous adjustment. Caffeine improves task-switching performance by increasing anticipatory processing, known as endogenous preparation. In the switching block, participants can accurately apply the task rule only when the stimulus is present, which primarily relies on exogenous adjustment [56,60]. This cognitive difference may have also influenced the results. Additionally, all participants in this study were healthy female university students with good cognitive function, and in the non-switching blocks of the task, participants only need to respond based on a single rule. Considering the relatively low difficulty level, there may be a ceiling effect. Thus, these factors may potentially influence the enhancing effects of tDCS and caffeine, leading to differences from the results of previous studies. Since we did not observe any improvement in cognitive flexibility from the individual interventions, it is understandable that the combined intervention also failed to produce a positive effect.

Several limitations should be acknowledged. Firstly, the intervention doses used were based on the known effects of tDCS and caffeine alone on cognitive functions. However, these may not be optimal for their combined use. Future studies should systematically explore the effects of different dose combinations of tDCS and caffeine (e.g. with body weight-adjusted caffeine) on cognitive functions to identify the optimal dose. Secondly, as an exploratory study, future research will need to employ methods such as electroencephalography (EEG) to validate the inferences regarding the underlying mechanisms of the combined intervention in this experiment. Thirdly, since all participants in the study were female, and considering the sex differences in the effects of caffeine, the experimental results should be generalized with caution. Lastly, our study did not verify the integrity of blinding protocol or successful caffeine abstinence compliance, which may have potentially compromised the validity of the experimental results.

## 5. Conclusions

In summary, the present study demonstrates that the combination of tDCS and caffeine has beneficial effects on cognitive function, which are more significant and short-term sustained compared to either intervention alone. We speculate that the improvement in cortical excitability may be the mechanism underlying this enhancement in cognitive function. These findings suggest novel avenues for enhancing cognitive function in healthy individuals.

## Data Availability

Data will be provided upon request.

## References

[1] Miller EK, Cohen JD. An integrative theory of prefrontal cortex function. Annu Rev Neurosci. 2001;24(1):167–13. doi: 10.1146/annurev.neuro.24.1.167 11283309

[2] Diamond A. Executive functions. Annu Rev Psychol. 2013;64(1):135–168. doi: 10.1146/annurev-psych-113011-143750 23020641 PMC4084861

[3] Baddeley A. Working memory. Curr Biol. 2010;20(4):R136–R140. doi: 10.1016/j.cub.2009.12.014 20178752

[4] Miyake A, Friedman NP, Emerson MJ, et al. The unity and diversity of executive functions and their contributions to complex “frontal lobe” tasks: a latent variable analysis. Cogn Psychol. 2000;41(1):49–100. doi: 10.1006/cogp.1999.0734 10945922

[5] Davidson MC, Amso D, Anderson LC, et al. Development of cognitive control and executive functions from 4 to 13 years: evidence from manipulations of memory, inhibition, and task switching. Neuropsychologia. 2006;44(11):2037–2078. doi: 10.1016/j.neuropsychologia.2006.02.006 16580701 PMC1513793

[6] Niendam TA, Laird AR, Ray KL, et al. Meta-analytic evidence for a superordinate cognitive control network subserving diverse executive functions. Cognit Affect Behav Neurosci. 2012;12(2):241–268. doi: 10.3758/s13415-011-0083-5 22282036 PMC3660731

[7] Dresler M, Sandberg A, Bublitz C, et al. Hacking the brain: dimensions of cognitive enhancement. ACS Chem Neurosci. 2018;10(3):1137–1148. doi: 10.1021/acschemneuro.8b00571 PMC6429408 30550256

[8] Nitsche MA, Fricke K, Henschke U, et al. Pharmacological modulation of cortical excitability shifts induced by transcranial direct current stimulation in humans. J Physiol. 2003;553(1):293–301. doi: 10.1113/jphysiol.2003.049916 12949224 PMC2343495

[9] Nitsche MA, Paulus W. Sustained excitability elevations induced by transcranial DC motor cortex stimulation in humans. Neurology. 2001;57(10):1899–1901. doi: 10.1212/WNL.57.10.1899 11723286

[10] Hoy KE, Emonson MRL, Arnold SL, et al. Testing the limits: investigating the effect of tDCS dose on working memory enhancement in healthy controls. Neuropsychologia. 2013;51(9):1777–1784. doi: 10.1016/j.neuropsychologia.2013.05.018 23751169

[11] Loftus AM, Yalcin O, Baughman FD, et al. The impact of transcranial direct current stimulation on inhibitory control in young adults. Brain Behav. 2015;5(5). doi: 10.1002/brb3.332 PMC4389055 25874165

[12] Yue T, Liu L, Nitsche MA, et al. Effects of high-intensity interval training combined with dual-site transcranial direct current stimulation on inhibitory control and working memory in healthy adults. Hum Mov Sci. 2024;96:103240. doi: 10.1016/j.humov.2024.103240 38875731

[13] Ke Y, Wang N, Du J, et al. The effects of transcranial direct current stimulation (tDCS) on working memory training in healthy young adults. Front Hum Neurosci. 2019;13. doi: 10.3389/fnhum.2019.00019 PMC6367257 30774590

[14] Harland BF. Caffeine and nutrition. Nutrition. 2000;16(7–8):522–526. doi: 10.1016/S0899-9007(00)00369-5 10906543

[15] Lorist MM, Tops M. Caffeine, fatigue, and cognition. Brain Cogn. 2003;53(1):82–94. doi: 10.1016/S0278-2626(03)00206-9 14572506

[16] Isokawa M. Caffeine-induced suppression of GABAergic inhibition and calcium-independent metaplasticity. Neural Plast. 2016;2016:1–7. doi: 10.1155/2016/1239629 PMC4779589 26998364

[17] Kerkhofs A, Xavier AC, da Silva BS, et al. Caffeine controls glutamatergic synaptic transmission and pyramidal neuron excitability in human neocortex. Front Pharmacol. 2018;8. doi: 10.3389/fphar.2017.00899 PMC5758559 29354052

[18] Brunyé TT, Mahoney CR, Lieberman HR, et al. Caffeine modulates attention network function. Brain Cogn. 2010;72(2):181–188. doi: 10.1016/j.bandc.2009.07.013 19733954

[19] Haskell CF, Kennedy DO, Wesnes KA, et al. Cognitive and mood improvements of caffeine in habitual consumers and habitual non-consumers of caffeine. Psychopharmacol (Berl). 2005;179(4):813–825. doi: 10.1007/s00213-004-2104-3 15678363

[20] Lattari E, Vieira LAF, Oliveira BRR, et al. Effects of transcranial direct current stimulation with caffeine intake on muscular strength and perceived exertion. J Strength Cond Res. 2019;33(5):1237–1243. doi: 10.1519/JSC.0000000000003123 30908367

[21] Lattari E, Vieira LAF, Santos LER, et al. Transcranial direct current stimulation combined with or without caffeine: effects on training volume and pain perception. Res Q Exerc Sport. 2022;94(1):45–54. doi: 10.1080/02701367.2021.1939251 35025723

[22] Domaszewski P. Gender differences in the frequency of positive and negative effects after acute caffeine consumption. Nutrients. 2023;15(6):1318. doi: 10.3390/nu15061318 36986044 PMC10052698

[23] Wilhelmus MMM, Hay JL, Zuiker RGJA, et al. Effects of a single, oral 60 mg caffeine dose on attention in healthy adult subjects. J Psychopharmacol. 2016;31(2):222–232. doi: 10.1177/0269881116668593 27649778

[24] Tian S, Mou H, Fang Q, et al. Comparison of the sustainability effects of high-intensity interval exercise and moderate-intensity continuous exercise on cognitive flexibility. Int J Environ Res Public Health. 2021;18(18):9631. doi: 10.3390/ijerph18189631 34574554 PMC8467653

[25] Jan Y-K, Zhang Z, Zhang B, et al. The effects of using an active workstation on executive function in Chinese college students. PLOS ONE. 2018;13(6):e0197740. doi: 10.1371/journal.pone.0197740 29879124 PMC5991683

[26] Wang J, Tian J, Hao R, et al. Transcranial direct current stimulation over the right DLPFC selectively modulates subprocesses in working memory. PeerJ. 2018;6:6. doi: 10.7717/peerj.4906 PMC5978386 29868292

[27] Liguori A. Absorption and subjective effects of caffeine from coffee, cola and capsules. Pharmacol Biochem Behav. 1997;58(3):721–726. doi: 10.1016/S0091-3057(97)00003-8 9329065

[28] Friehs MA, Frings C. Offline beats online. Neuroreport. 2019;30(12):795–799. doi: 10.1097/WNR.0000000000001272 31283711

[29] Lu H, Liu Q, Guo Z, et al. Modulation of repeated anodal HD-tDCS on attention in healthy young adults. Front psychol. 2020;11:11. doi: 10.3389/fpsyg.2020.564447 33329194 PMC7714753

[30] Fernandes TP, Zhang W, Liu H, et al. Immediate and short-term effects of single-task and motor-cognitive dual-task on executive function. PLOS ONE. 2023;18(8):e0290171. doi: 10.1371/journal.pone.0290171 37585447 PMC10431647

[31] Schwippel T, Papazova I, Strube W, et al. Beneficial effects of anodal transcranial direct current stimulation (tDCS) on spatial working memory in patients with schizophrenia. Eur Neuropsychopharmacol. 2018;28(12):1339–1350. doi: 10.1016/j.euroneuro.2018.09.009 30292415

[32] Papazova I, Strube W, Becker B, et al. Improving working memory in schizophrenia: effects of 1 mA and 2 mA transcranial direct current stimulation to the left DLPFC. Schizophr Res. 2018;202:203–209. doi: 10.1016/j.schres.2018.06.032 29954701

[33] Stagg CJ, Nitsche MA. Physiological basis of transcranial direct current stimulation. Neurosci. 2011;17(1):37–53. doi: 10.1177/1073858410386614 21343407

[34] Ohn SH, Park C-I, Yoo W-K, et al. Time-dependent effect of transcranial direct current stimulation on the enhancement of working memory. Neuroreport. 2008;19(1):43–47. doi: 10.1097/WNR.0b013e3282f2adfd 18281890

[35] Ribeiro JA, Sebastião AM, Cunha RA, et al. Caffeine and adenosine. J Alzheimer’s Disease. 2010;20(s1):S3–S15. doi: 10.3233/JAD-2010-1379 20164566

[36] McLellan TM, Caldwell JA, Lieberman HR. A review of caffeine’s effects on cognitive, physical and occupational performance. Neurosci Biobehav Rev. 2016;71:294–312. doi: 10.1016/j.neubiorev.2016.09.001 27612937

[37] Einöther SJL, Giesbrecht T. Caffeine as an attention enhancer: reviewing existing assumptions. Psychopharmacol (Berl). 2012;225(2):251–274. doi: 10.1007/s00213-012-2917-4 23241646

[38] Fredholm BB, Bättig K, Holmén J, et al. Actions of caffeine in the brain with special reference to factors that contribute to its widespread use. Pharmacol Rev. 1999;51(1):83–133. doi: 10.1016/S0031-6997(24)01396-6 10049999

[39] Owen GN, Parnell H, De Bruin EA, et al. The combined effects of L-theanine and caffeine on cognitive performance and mood. Nutr Neurosci. 2013;11(4):193–198. doi: 10.1179/147683008X301513 18681988

[40] Cerqueira V, de Mendonça A, Minez A, et al. Does caffeine modify corticomotor excitability? Neurophysiologie clinique/Clin Neurophysiol. 2006;36(4):219–226. doi: 10.1016/j.neucli.2006.08.005 17095411

[41] Tremblay S, Beaulé V, Lepage J-F, et al. Anodal transcranial direct current stimulation modulates GABAB-related intracortical inhibition in the M1 of healthy individuals. Neuroreport. 2013;24(1):46–50. doi: 10.1097/WNR.0b013e32835c36b8 23196416

[42] Stagg CJ, Best JG, Stephenson MC, et al. Polarity-sensitive modulation of cortical neurotransmitters by transcranial stimulation. J Neurosci. 2009;29(16):5202–5206. doi: 10.1523/JNEUROSCI.4432-08.2009 19386916 PMC6665468

[43] Zulkifly MFM, Merkohitaj O, Brockmöller J, et al. Confounding effects of caffeine on neuroplasticity induced by transcranial alternating current stimulation and paired associative stimulation. Clin Neurophysiol. 2021;132(6):1367–1379. doi: 10.1016/j.clinph.2021.01.024 33762129

[44] Jacobson L, Koslowsky M, Lavidor M. tDCS polarity effects in motor and cognitive domains: a meta-analytical review. Exp Brain Res. 2011;216(1):1–10. doi: 10.1007/s00221-011-2891-9 21989847

[45] Wang C, Zhu Y, Dong C, et al. Effects of various doses of caffeine ingestion on intermittent exercise performance and cognition. Brain Sci. 2020;10(9):595. doi: 10.3390/brainsci10090595 32872249 PMC7564618

[46] Perrotta D, Bianco V, Berchicci M, et al. Anodal tDCS over the dorsolateral prefrontal cortex reduces Stroop errors. A comparison of different tasks and designs. Behav Brain Res. 2021;405:405. doi: 10.1016/j.bbr.2021.113215 33662440

[47] Naros G, Geyer M, Koch S, et al. Enhanced motor learning with bilateral transcranial direct current stimulation: impact of polarity or current flow direction? Clin Neurophysiol. 2016;127(4):2119–2126. doi: 10.1016/j.clinph.2015.12.020 26818881

[48] Vines BW, Cerruti C, Schlaug G. Dual-hemisphere tDCS facilitates greater improvements for healthy subjects’ non-dominant hand compared to uni-hemisphere stimulation. BMC neurosci. 2008;9(1). doi: 10.1186/1471-2202-9-103 PMC2584652 18957075

[49] Fujimoto S, Yamaguchi T, Otaka Y, et al. Dual-hemisphere transcranial direct current stimulation improves performance in a tactile spatial discrimination task. Clin Neurophysiol. 2014;125(8):1669–1674. doi: 10.1016/j.clinph.2013.12.100 24411524

[50] McIntire LK, McKinley RA, Nelson JM, et al. Transcranial direct current stimulation versus caffeine as a fatigue countermeasure. Brain Stimul. 2017;10(6):1070–1078. doi: 10.1016/j.brs.2017.08.005 28851554

[51] Dixit A, Goyal A, Thawani R, et al. Effect of caffeine on information processing: evidence from Stroop task. Indian J Psychol Med. 2012;34(3):218–222. doi: 10.4103/0253-7176.106013 23441060 PMC3573570

[52] Zhang B, Liu Y, Wang X, et al. Cognition and brain activation in response to various doses of caffeine: a near-infrared spectroscopy study. Front psychol. 2020;11:11. doi: 10.3389/fpsyg.2020.01393 32719638 PMC7350703

[53] Edwards S, Brice C, Craig C, et al. Effects of caffeine, practice, and mode of presentation on Stroop task performance. Pharmacol Biochem Behav. 1996;54(2):309–315. doi: 10.1016/0091-3057(95)02116-7 8743589

[54] Yuan Y, Li G, Ren H, et al. Caffeine effect on cognitive function during a Stroop task: fNIRS study. Neural Plast. 2020;2020:1–8. doi: 10.1155/2020/8833134 PMC7700036 33273906

[55] Ali A, O’Donnell J, Von Hurst P, et al. Caffeine ingestion enhances perceptual responses during intermittent exercise in female team-game players. J Sports Sci. 2015;34(4):330–341. doi: 10.1080/02640414.2015.1052746 26045170

[56] Tieges Z, Snel J, Kok A, et al. Caffeine improves anticipatory processes in task switching. Biol psychol. 2006;73(2):101–113. doi: 10.1016/j.biopsycho.2005.12.005 16549227

[57] Tieges Z, Snel J, Kok A, et al. Effects of caffeine on anticipatory control processes: evidence from a cued task‐switch paradigm. Psychophysiology. 2007;44(4):561–578. doi: 10.1111/j.1469-8986.2007.00534.x 17539921

[58] Leite J, Carvalho S, Fregni F, et al. The effects of cross-hemispheric dorsolateral prefrontal cortex transcranial direct current stimulation (tDCS) on task switching. Brain Stimul. 2013;6(4):660–667. doi: 10.1016/j.brs.2012.10.006 23142550

[59] Luft CDB, Zioga I, Banissy MJ, et al. Relaxing learned constraints through cathodal tDCS on the left dorsolateral prefrontal cortex. Sci Rep. 2017;7(1). doi: 10.1038/s41598-017-03022-2 PMC5462743 28592845

[60] Wang Z, Zhu R, Rehman AU, et al. Dorsolateral prefrontal cortex and task-switching performance: effects of anodal transcranial direct current stimulation. Neuroscience. 2020;446:94–101. doi: 10.1016/j.neuroscience.2020.08.020 32858145

